# Changes in mental health of adolescents during three months of the COVID-19 pandemic: longitudinal study

**DOI:** 10.1192/bjo.2025.10801

**Published:** 2025-08-15

**Authors:** Matthias Pierce, Emily Banwell, Stephanie Gillibrand, Margarita Panayiotou, Pamela Qualter, Luke Munford, Ola Demkowicz

**Affiliations:** Centre for Women’s Mental Health, Division of Psychology and Mental Health, The University of Manchester, UK; Manchester Institute of Education, The University of Manchester, UK; Centre for Primary Care, School of Health Sciences, The University of Manchester, UK; Health Organisation, Policy and Economics, The University of Manchester, UK

**Keywords:** COVID-19, young people’s mental health, adolescence, hyperactivity, emotional problems

## Abstract

**Background:**

Understanding the effect(s) of the COVID-19 pandemic is key for planning for future pandemics.

**Aims:**

This study examines change in self-reported mental health difficulties during three months of the pandemic among adolescent (10- to 15-year-olds) participants from the UK Household Longitudinal Study (waves 7, 9 and 11 of the main survey and waves 4, 5 and 8 of the COVID-19 surveys).

**Method:**

We focused on mental health difficulties using the Strengths and Difficulties Questionnaire (SDQ), using repeated cross-sectional and longitudinal analyses to examine data among 6471 adolescents who responded to at least one survey since 2015, and 2,300 who responded to at least one COVID-19 survey during July 2020, November 2020 or March 2021.

**Results:**

Repeated cross-sectional data showed similar mean total SDQ across surveys before and during the pandemic (range during pandemic 11.4 to 11.9; range pre-pandemic 11.1 to 11.8). Longitudinal analyses provided no evidence of mental health change compared with pre-pandemic trends (estimated change mean SDQ (β) = 0.05, 95% CI −0.42 to 0.51; *p* = 0.85), or differential sociodemographic effects, except greater effects in rural households (β = 0.67, 95% CI −0.08 to 1.41) than urban environments (β = −0.18, 95% CI −0.69 to 0.33). Though subscales generally saw higher scores during the pandemic than before, these were consistent with pre-pandemic trends, excepting a slight improvement in conduct problems (β = −0.26, 95% CI 0.12 to 0.40).

**Conclusions:**

The study offers evidence among a representative sample that mental health difficulties did not, on average, deteriorate for adolescents during three months of the pandemic.

As governments enacted measures to curb infection from the COVID-19 virus, including restricting social contacts and partial school closures, there were widespread concerns that such measures would adversely affect mental health. Adolescents were considered particularly vulnerable, because social support is considered central to their well-being,^1^ and they have previously been found to be disproportionately affected by public emergencies.^2^ Understanding the effect(s) of the pandemic and pandemic-related policies and restrictions on adolescents is central to knowing whether we need ongoing targeted support for young people who were adolescents during the pandemic. This is especially crucial given increasing pressures on child and adolescent mental health services.^3^ In addition, a greater understanding of ways that the pandemic and pandemic-related policy decisions affected vulnerable groups ensures decision-makers are better prepared for any future pandemics and crises.

## Current evidence on adolescent mental health in the pandemic

Though initial evidence pointed towards a deterioration in the mental health of adolescents during the pandemic,^4^ more recent empirical studies have highlighted a more complex picture. In a follow-up sample of 1415 adolescents (11- to 16-year-olds) in England, the Mental Health of Children and Young People Survey reported a prevalence of ‘probable mental disorder’ of 17.7% in 2021, up from 13.3% in 2017, with symptoms relating to peer problems and hyperactivity particularly affected,^5^ though this study suffered from high levels of attrition (60%). Studies using self-reported data from children and adolescents have found higher depressive symptoms during the pandemic^6,7^ than before. However, it seems the evidence base is varied and, at times, contradictory. A review of 51 studies that included data from before and during the pandemic indicated a slight deterioration across adolescent mental health difficulties but, overall, a mixed evidence base with no clear pattern.^8^ Mansfield et al^9^ identified variations across mental health domains before and during the pandemic among a UK sample of adolescents, and found no overall effect of the pandemic on adolescent externalising difficulties but reported increased depressive symptoms and decreased life satisfaction. Guzman Holst et al^10^ indicated that most young people adapted well and experienced low stable symptoms in the pandemic in the UK context, and similarly Knowles et al^11^ reported no evidence of an overall increase in mental distress among adolescents in inner London mid-pandemic relative to pre-pandemic but did identify a range of subgroup effects.

Indeed, there is evidence to suggest uneven impacts of the pandemic among adolescents. Other studies report that the pandemic disproportionately affected children and young people with pre-existing vulnerabilities and on the basis of socioeconomic based inequalities and gender – that is, greater mental health impacts for girls and young women,^12^ for those from lower-income households,^13^ deprived areas,^12^ urban areas^14^ and English regions that are within Northern England,^15^ and for those with special educational needs (SEN) or neurodevelopmental conditions.^16^ Emerging evidence on children and young people from UK ethnic minority backgrounds suggests that their mental health was less affected than their White peers’. However, these studies often aggregate non-White groups, disallowing within group variation to be fully understood, and other studies are bound by sample size and specificity issues.^11^ Further, we hypothesise greater effects for those living in the North of England due to the disproportionate impacts of the pandemic on this region.^15^

Given this mixed evidence, ongoing efforts to disentangle effects are valuable; there is a need for ongoing research, particularly as there are serious limitations in much existing work that need to be addressed in future research. For example, much of the existing evidence is limited by a lack of pre-pandemic measures, reducing the ability to adjust for long-term trends in adolescent mental health, which was deteriorating for some years before the pandemic.^17^ Evidence indicating a more varied picture tends to include pre-pandemic measures, and thus further work exploring such data points is needed. In addition, many researchers have used convenience sampling strategies, such as questionnaires circulated on social media, that are likely to be susceptible to participation bias: those with the most significant mental health needs are often the least likely to complete surveys.^18^ Indeed, the above review of 51 studies capturing changes before and during the pandemic highlighted frequent quality issues, in particular questions regarding the representativeness of the sample.^8^

## Current study

To overcome the limitations of previous work, and to contribute to the development of a clearer picture of adolescent mental health post-COVID-19, the current study considers adolescent (11- to 15-year-olds) participants in the UK Household Longitudinal Study, a nationally representative cohort, to examine changes to adolescents’ self-reported mental health difficulties during three months of the pandemic: July 2020, September 2020 and March 2021. Data comes from three data collection waves before the pandemic (waves 7, 9 and 11 of the main survey and waves 4, 5 and 8 of the COVID-19 surveys), allowing for modelling of pre-pandemic trends, and three COVID-19 waves, enabling the assessment of the pandemic’s impact during different phases of the pandemic. These data are available to anybody registered with the UK data service. We use a mixture of repeated cross-sectional and longitudinal analysis. We hypothesised that the pandemic led to a deterioration in various mental health dimensions among adolescents, particularly during the summer of 2020 and for girls, those in deprived households, ethnic minorities, those in urban environments and those in the north of England.

## Method

### UK household longitudinal study data

**Fig. 1:**
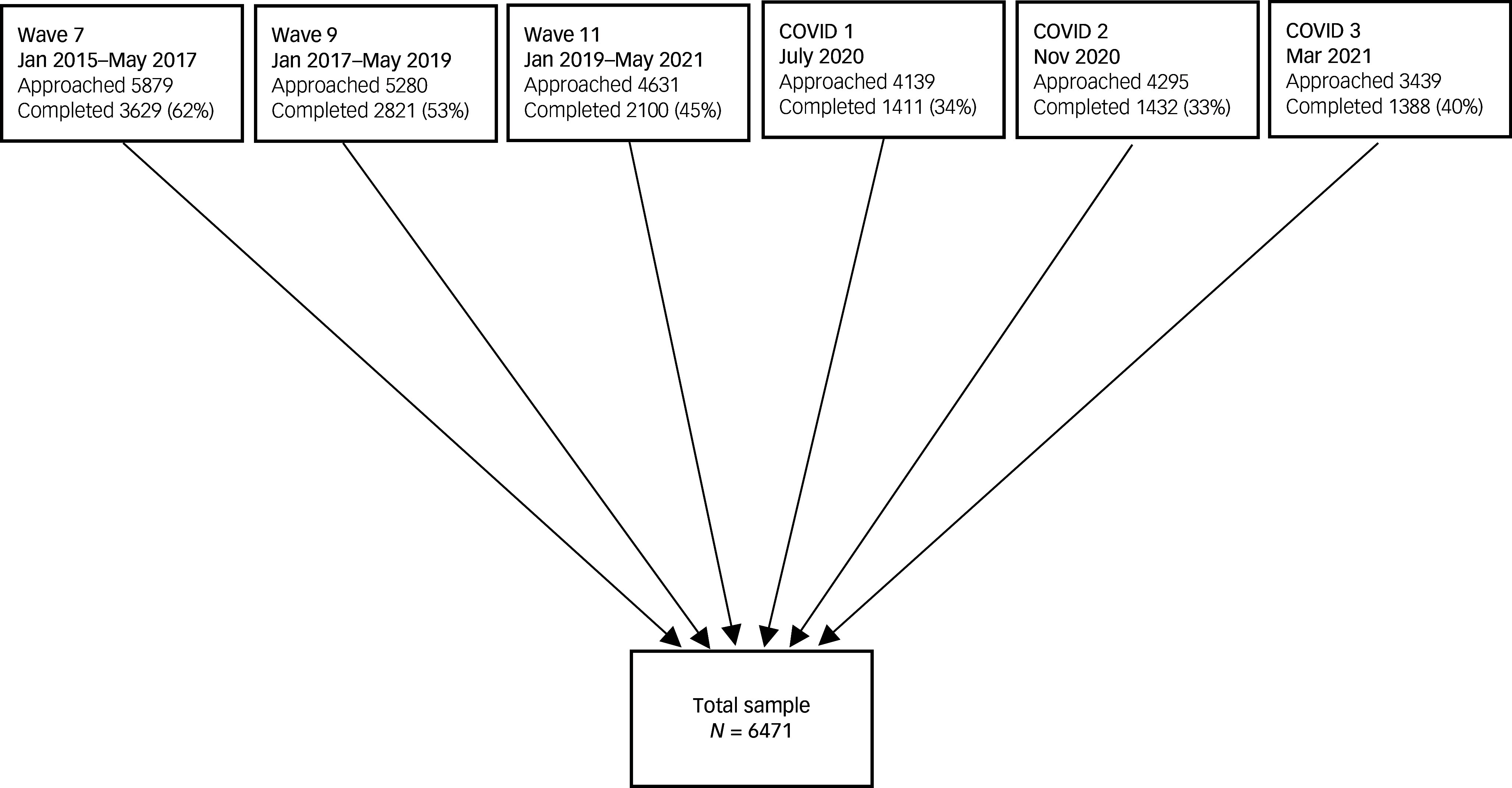
Flow diagram showing selection of the cohort, UK adolescent (aged 10–15) respondents to the UK Household Longitudinal Study. Note that adolescents may respond to multiple waves and therefore the total sample size is smaller than the sum of individual waves.

We used data from 2015 to 2021 from the main survey and the COVID-19 survey of the UK Household Longitudinal Study (UKHLS; also called Understanding Society), a large survey of roughly 40 000 UK households, probability sampled and including a boosted sample from ethnic minority groups to account for the historic underrepresentation of these groups in surveys. The main yearly survey includes a ‘youth’ self-completion survey for all people in each household aged 10 to 15 years, which includes questions about their mental health every two years. Successive waves include overlapping but distinct samples as individuals in the household enter and exit the age range. Young people were offered a £5 voucher for partial completion of the survey. In addition, specific COVID-19 questionnaires were issued during the pandemic to all households who participated in the two most recent pre-COVID-19 surveys. Young people were asked about their mental health during three COVID-19 surveys in July 2020, November 2020 and March 2021. The sample included all those aged 10–15 who responded to at least one survey from 2015 where mental health was measured using the Strengths and Difficulties Questionnaire (SDQ) (waves 7, 9 and 11 of the main survey and waves 4, 5 and 8 of the COVID-19 surveys). The sample included 6471 young people from 4465 families (see Fig. 1).

The data controllers provide weights for each wave in the data-set.^19^ These are intended to correct for the stratified sampling design, as well as non-response, and are calibrated to official population statistics. Non-response probability was estimated using a backwards, stepwise logistic regression model based on predictors including variables from the sampling frame (e.g. sample month and geographical region), predicted ethnic density at the postcode sector level and a range of indicators obtained from national census and neighbourhood statistics data. The calibration step ensures that the weighted sample is representative of the UK population.

Young people’s mental health was measured using self-response to the SDQ.^20^ The SDQ is a short behavioural and emotional screening questionnaire for children and young people, comprising five subscales: emotional problems, conduct problems, hyperactivity/inattention, peer relationship problems and pro-social behaviour. Each subscale comprises five items (totalling 25 items), and participants respond using a three-point scale to reflect on how items apply to them over the last six months: 0 = ‘not true’, 1 = ‘somewhat true’ and 2 = ‘certainly true’. Scores can be summed within each individual subscale, where a higher score indicates greater level of difficulty, excepting pro-social behaviour, which functions in the opposite orientation. The four difficulty-focused subscales (excluding pro-social behaviour) can be summed to create a total difficulties score. Its brevity allows efficient reporting on multiple mental health facets without use of more exhaustive diagnostic tools, though this does limit the range of symptoms it can capture. The English-language self-report version of the SDQ has demonstrated acceptable reliability, and criterion validity against emotional and behavioural disorders.^21^

Covariates were also extracted on gender,^a^ age at interview, ethnicity (Asian, Black, Mixed, White, Other) and region of the UK (Northern England, Midlands, Southern England, Wales, Scotland, Northern Ireland). It should be noted that ethnicity was not recorded in the COVID-19 waves so was imputed from prior waves using the latest available measure. In addition, the total net household monthly income was determined using parent data, measured at wave 11 and categorised according to the quintile across the whole sample.

### Statistical analysis

Two analyses were conducted: the first described trends using repeated cross-sectional data; the second considered changes using longitudinal data. For the cross-sectional analysis, mean SDQ scores (total and individual subscales) were calculated for each half year (January to June, July to December) from 2015 to 2019 and for each subsequent COVID-19 survey. It was decided to display these by half year to show changes that could occur *within* years and to ensure that the sample size was sufficient for precise estimates. Statistics were estimated after applying cross-sectional survey weights. For the longitudinal analysis, generalised estimating equations (GEE) were fitted to repeated measures of SDQ, with an indicator variable for the ‘pandemic’ set to one if the data point was after 16 March 2020, and zero otherwise. The pandemic included both those in the COVID-19-specific surveys and those reporting to wave 11 during this period (*n* = 670). These models controlled for calendar time of the interview (parameterised as years since the first interview in the sample, i.e. years since 1 January 2015), calendar time squared, an indicator for the month of the interview and an interaction between calendar time and gender. The regression of this variable on SDQ therefore addressed the hypothesis that symptoms worsened following the start of the pandemic, accounting for historical trends. These used weights from wave 11.

Interactions were fitted to test whether change in overall SDQ associated with the pandemic differed by subgroups of gender, age (10–13 as early adolescence, 14–15 years as moving into mid-adolescence), ethnicity, region of the UK, quintile of household deprivation and period of the pandemic (July 2020, November 2020 or March 2021). Additional regression models examined the effect of the pandemic on domains of the SDQ (emotional, conduct, hyperactivity, peer problems, pro-social). Due to the truncated nature of these measures, these models were parameterised as Poisson GEE models, with the identity link function.

Data were missing for any variable for 12% of measurements (see Supplementary Appendix A, available at https://doi.org/10.1192/bjo.2025.10801). Multiple imputation was used to account for potential departures from missing completely at random assumptions, using the chained equations method to create ten multiply imputed data-sets. Multiple imputation models included all SDQ domains, as well as sociodemographic variables of gender, age, region, income, ethnicity and an urban/rural indicator.

The analysis plan was pre-specified before analysis (see Supplementary Appendix B). On the suggestion of a reviewer, we added the time-updated covariate ‘age’ to the model. We added an additional sensitivity analysis, after the analysis plan was written, whereby the item ‘I am usually on my own. I generally play alone or keep to myself’ was removed, because it was considered this may have a different meaning during the first year of the pandemic. We also explored whether SDQ subscales measured similar latent constructs before and during the COVID-19 pandemic by fitting longitudinal factor invariance models, using the last pre-COVID-19 survey and the first COVID-19 survey (see Supplementary Appendix C). All data management and analyses were conducted using Stata version 14 (StataCorp, College Station, TX, USA; www.stata.com). Plots were produced using the ggplot2 package version 3.4.4 (The R Foundation for Statistical Computing, Vienna, Austria; www.r-project.org), running on R version 4.3.3 (Windows; The R Foundation, Vienna, Austria; www.r-project.org). Psychometric analyses were conducted using Mplus version 8 (Muthén & Muthén, Los Angeles, CA, USA; www.statmodel.com).

## Results

The cohort consisted of 6471 adolescents (aged 10–15) sampled over six waves of data collection from January 2015 to March 2021, including 2300 who completed at least one survey during the pandemic waves of data collection. The characteristics of those who completed a pandemic survey were similar to the whole sample (Table 1). Half were girls and the mean age at their first completed wave was 13.0 years (s.d. = 1.76). Adolescents completed a mean of 1.98 waves of data collection and their median follow-up time was 120 days (interquartile range 0 to 763).

**Table 1:** Unweighted and weighted sample characteristics for adolescent (aged 10–15) respondents to the UK Household Longitudinal Study

Characteristics	Whole sample, *N* = 6471			COVID-19 data, *N* = 2300		
	%^a^			%^a^		
	*n*	Unweighted	Weighted^b^	*n*	Unweighted	Weighted^b^
Gender^c^						
Girl	3222	49.8	49.4	1159	50.4	48.5
Boy	3219	49.7	50.6	1113	48.4	51.5
Missing	30	0.5		28	1.2	
Region						
Northern England	1540	23.8	23.1	534	23.2	21.9
Midlands	1013	15.7	16.5	363	15.8	17.4
Southern England	2716	42.0	45.3	969	42.1	44.2
Wales	294	4.5	3.8	107	4.7	4.1
Scotland	493	7.6	8.4	168	7.3	9.3
Northern Ireland	363	5.6	3.0	108	4.7	3.1
Missing	52	0.8		51	2.2	
Ethnicity						
White	4040	62.4	84.4	1167	50.7	82.9
Asian	949	14.7	6.5	253	11.0	6.8
Black	327	5.1	3.4	45	2.0	2.9
Other	459	7.1	5.7	163	7.1	7.5
Missing	696	10.8		672	29.2	
Household income quintile						
Lowest	1607	24.8	25.5	557	24.2	26.5
2nd	1517	23.4	28.5	530	23.0	29.9
3rd	1257	19.4	19.9	464	20.2	18.9
4th	1024	15.8	16.1	424	18.4	15.4
Highest	698	10.8	10.0	300	13.0	9.2
Missing	368	5.7		25	1.1	
Urban or rural						
Urban	5008	77.4	75.8	1740	75.7	75.3
Rural	1398	21.6	24.2	504	21.9	24.7
Missing	65	1.0		56	2.4	

a. Column percentages.

b. Excluding missing category.

c. Our gender variable here relies on an imperfect proxy by drawing on self-reported binary sex data; however, in the absence of more inclusive gender data we sought to be sensitive to the ethical ramifications of implying attributions to sex and biological difference.

**Fig. 2:**
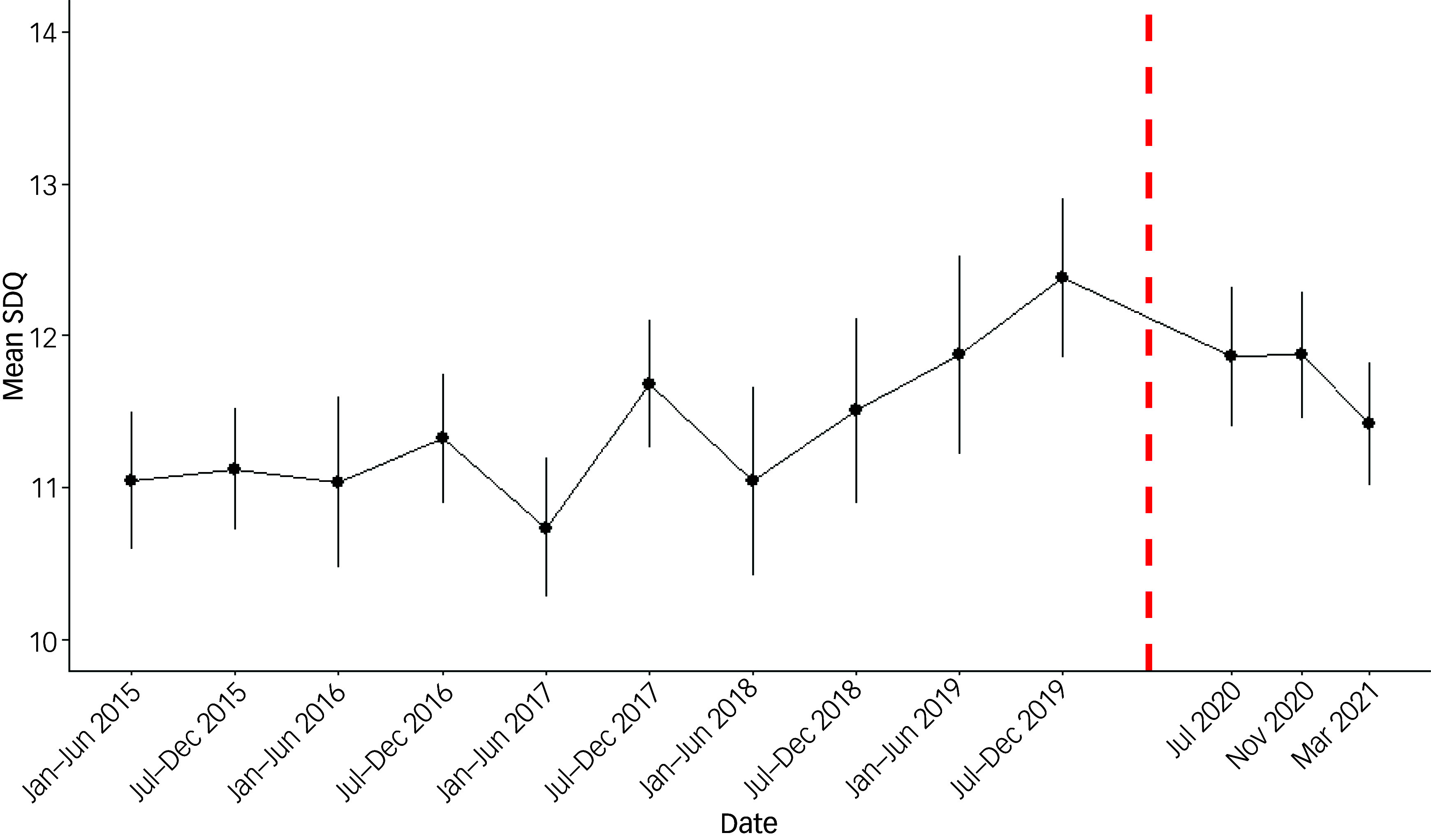
Survey-weighted mean Strengths and Difficulties 167Questionnaire (SDQ) since 2015 for adolescent (aged 10–15) respondents to the UK Household Longitudinal Study. Wave-specific values. The dashed line represents when the first government lockdown was announced (23 March 2020).

**Fig. 3:**
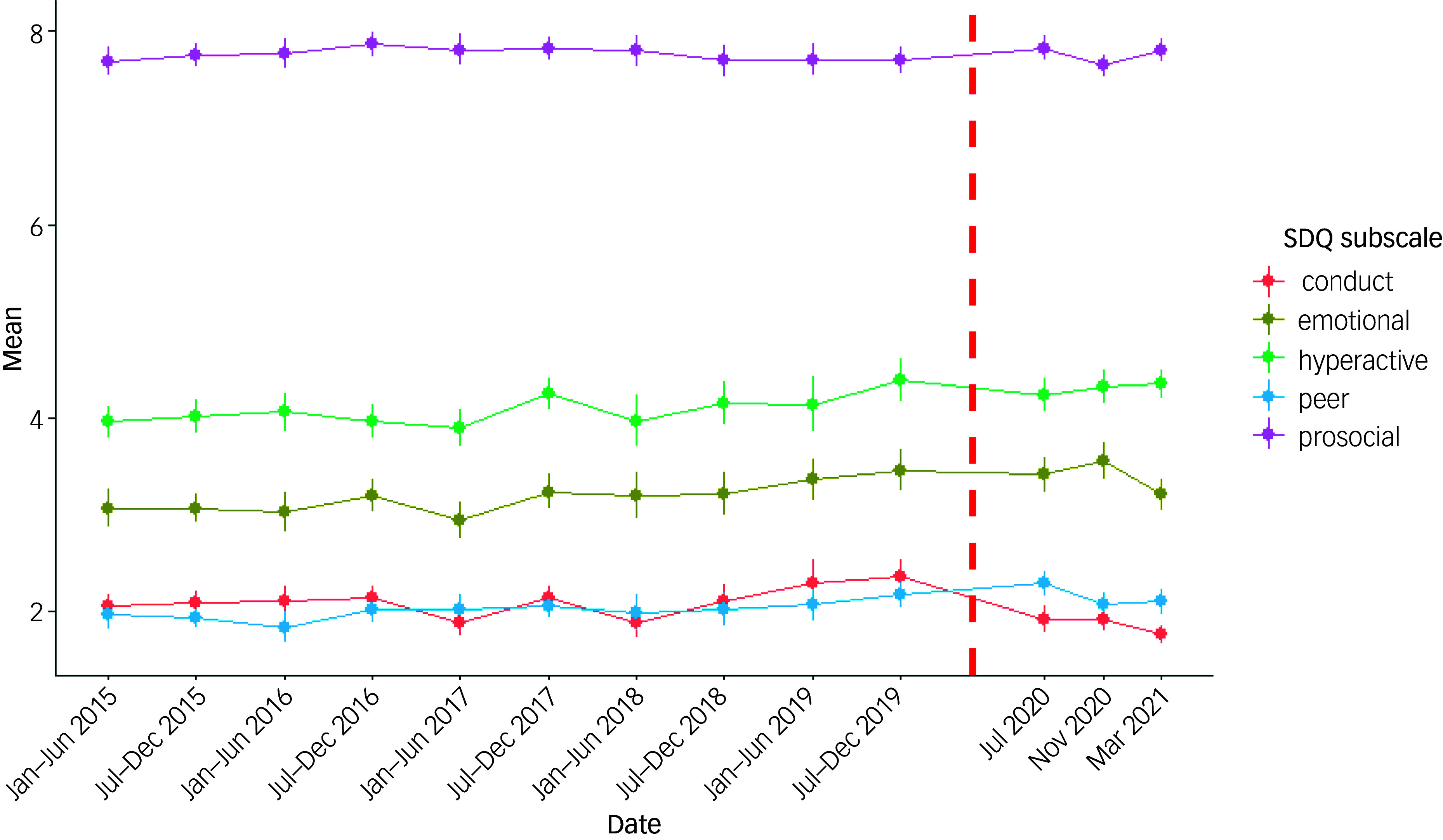
Survey-weighted mean Strengths and Difficulties 167Questionnaire (SDQ) subscales since 2015 for adolescent (aged 10–15) respondents to the UK Household Longitudinal Study. The dashed line represents when the first government lockdown was announced (23 March 2020).

Mean SDQ scores from 2015 to 2019 and for each subsequent COVID-19 survey are graphically displayed for the whole sample (see Fig. 2), then by SDQ domains (see Fig. 3).

Results from the repeated cross-sectional data show that the mean total SDQ reported during each COVID-19 survey (taken during July 2020, November 2020 and March 2021) was similar to that reported in the pre-COVID surveys (range in weighted mean SDQ during pandemic 11.4 to 11.9; range pre-pandemic 11.1 to 11.8; see Supplementary Appendix D). There was a small increase in the s.d. of the total SDQ during the pandemic, e.g. from 6.1 in the last pre-pandemic survey to 6.3 in the March 2021 survey (Supplementary Appendix D). Inspection of graphs of total SDQ, and SDQ by sub-domain, indicates no obvious departures from pre-pandemic trends (Figs 2 and 3).

Results from the GEE models (see Table 2) provided no evidence that mental health changed during the pandemic, compared with pre-pandemic trends (estimated change in mean SDQ (β) = 0.06, 95% CI −0.41 to 0.52; *p* = 0.81). There was also no evidence of a differential effect of the pandemic by gender (*p* = 0.13), age (*p* = 0.09), ethnicity (*p* = 0.96), region of UK (*p* = 0.33) or household income (*p* = 0.36). There was slight evidence that of adolescents living in an urban or rural household (*p* = 0.04), those in rural households were more detrimentally affected (β = 0.65, 95% CI −0.09 to 1.38) than those in urban environments (β = −0.16, 95% CI −0.67 to 0.35; however, this did not cross the pre-specified threshold for significance of *p* = 0.01.

**Table 2:** Estimated change in mean total SDQ, overall and by subgroups from generalised estimating equation (GEE) models for adolescent (aged 10−15) respondents to the UK Household Longitudinal Study

Group	*N* (%)^a^	Mean SDQ score		Change (β)^b^	*p*-value^c^
		Pre-pandemic	Pandemic		
All	6471 (100)	11.35	11.73	0.06 [−0.41, 0.52]	0.811
Girls	3224 (50.0)	11.45	12.26	0.37 [−0.22, 0.97]	0.132
Boys	3217 (50.0)	11.25	11.20	−0.24 [−0.88, 0.39]	
10−12 years	2323 (36.4)	11.16	11.35	−0.31 [−0.97, 0.36]	0.091
13−15 years	4058 (63.6)	11.41	12.10	0.27 [−0.23, 0.76]	
White	4038 (69.9)	11.56	11.95	0.05 [−0.45, 0.55]	0.959
Asian	949 (16,4)	9.44	9.32	0.05 [−0.45, 0.55]	
Black	327 (5.7)	9.44	10.93	0.20 [−0.74, 1.13]	
Other	461 (8.0)	10.81	10.92	0.40 [−1.20, 2.00]	
Northern England	1539 (24.0)	11.54	12.45	0.46 [−0.27, 1.19]	0.334
Midlands	1013 (15.8)	11.36	11.67	0.06 [−0.72, 0.84]	
Southern England	2716 (42.3)	11.35	11.43	−0.35 [−0.95, 0.26]	
Wales	295 (4.6)	10.74	11.08	1.17 [−0.50, 2.83]	
Scotland	493 (7.7)	11.22	11.89	0.34 [−0.85, 1.53]	
Northern Ireland	363 (5.7)	11.01	10.57	0.02 [−1.61, 1.64]	
Lowest income quintile	1607 (26.3)	11.83	11.42	0.08 [−0.66, 0.81]	0.351
2nd quintile	1517 (24.9)	12.00	12.78	0.09 [−0.64, 0.82]	
3rd quintile	1257 (20.6)	11.02	12.48	0.59 [−0.20, 1.39]	
4th quintile	1024 (16.8)	10.47	10.56	−0.14 [−0.86, 0.59]	
Highest income quintile	698 (11.4)	10.40	9.86	−0.45 [−1.22, 0.32]	
Urban	5008 (78.2)	11.31	11.56	−0.16 [−0.67, 0.35]	0.045
Rural	1398 (21.8)	11.48	12.22	0.65 [−0.09, 1.38]	

SDQ, Strengths and Difficulties Questionnaire.

a. *N* relates to the number of adolescents in each group prior to multiple imputation.

b. The beta coefficient relates to the estimated change from the GEE model, adjusting for calendar time, calendar time squared and age, and the interaction between calendar time and sex.

c. For the first test (i.e. ‘All’) the *p*-value tests the null hypothesis that the beta coefficient is zero. For all others, the *p*-value tests the null hypothesis that the beta coefficients across strata are equal (i.e. tests for effect modification).

Examining subscales of the SDQ, most saw higher scores during the pandemic than before, but these seemed to be in keeping with pre-pandemic trends (see Table 3). The exceptions were scores on the conduct problems subscale, where there was a slight improvement, reflected in the GEE model, which estimated scores that were 0.26 lower (95% CI 0.12 to 0.39). There was no evidence of a change in emotional problems (*p* = 0.77), hyperactivity (*p* = 0.27), peer problems (*p* = 0.29) or pro-social scores (*p* = 0.22).

**Table 3:** Estimated change in mean Strengths and Difficulties 167Questionnaire subscale from generalised estimating equation (GEE) models for adolescent (aged 10–15) respondents to the UK Household Longitudinal Study

Subscale	Mean score		Change (β)^a^	*p*-value
	Pre-pandemic	Pandemic		
Emotional	3.17	3.42	0.03 [−0.17, 0.23]	0.772
Conduct	2.11	1.88	−0.26 [−0.39, −0.12]	<0.001
Hyperactivity	4.07	4.29	0.11 [−0.08, 0.30]	0.266
Peer problems	2.00	2.13	0.09 [−0.07, 0.24]	0.290
Pro-social	7.76	7.75	−0.09 [−0.24, 0.05]	0.216

a. The beta-coefficient relates to the estimated change from the GEE model, adjusting for calendar time, calendar time squared and age, and the interaction between calendar time and sex.

Almost all subscales showed good longitudinal invariance properties between pre-pandemic and the first pandemic wave as evidenced by models with factor invariance structures not being judged to be a worse fit for the data than models without (see Supplementary Appendix C). The exception was the hyperactivity scale, where there was insufficient evidence to suggest that the factor loadings were consistent across time. In the sensitivity analysis, removing the item in the peer problem subscale ‘I am usually on my own. I generally play alone or keep to myself’ made little difference to the estimated effect of the pandemic.

## Discussion

The current study indicates little to no change in young people’s mental health associated with the pandemic across July 2020, November 2020 and March 2021 among a representative UK sample, when controlling for pre-pandemic trends. Specifically, we found no support for our hypothesis that the COVID-19 pandemic period was associated with a deterioration in mental health difficulties among adolescents; instead, no overall change in mental health difficulties was evidenced, except for a small *reduction* in self-reported conduct problems. Analysis also did not provide support for significant differences in impact across specific groups, including girls, those in deprived households and those in the north of England. These results diverge from the wider evidence base in several ways (8–11) and offer an important contribution to knowledge and understanding of the effects of the pandemic for adolescent mental health.

### Stability of mental health across the pandemic

Our results indicate no significant changes across total mental health difficulties for adolescents aged 10–15 over three months of the pandemic, nor changes for most specific mental health domains. This is an important contribution given the currently mixed picture regarding adolescent mental health in the pandemic, with considerable variation across studies and mental health domains.^8,9,11^ There are several possible explanations for understanding how our study sits within that wider literature base. First, much existing evidence is limited by a lack of pre-pandemic measures and thus cannot capture the existing time trends in young people’s mental health.^17^ Without capturing those trends, the counterfactual ‘what would the mental health be if the pandemic had not happened?’ will be biased towards better mental health, and the estimated effect of the pandemic will be exaggerated. Indeed, the more varied findings already identified suggesting a more complex picture of mental health impact do tend to be those studies that, as in the current study, have incorporated pre-pandemic data.^8^ Second, studies often use non-probability samples, such as those obtained from social media, that are likely to be less representative of the population than probability samples,^18^ such as the one used here. Even among studies that incorporate data from both before and during the pandemic, issues of sampling and representativeness issues are common.^8^ The use of self-report data may also be a consideration, although this too diverges from the literature; interestingly, Newlove et al^8^ report that their meta-analysis of self-reported emotional difficulties indicated evidence of deterioration in emotional difficulties, whereas a corresponding meta-analysis of parent report found no such evidence.

A fourth consideration is the specific time points of data collection in this data-set: July 2020, November 2020 and March 2021. In the UK, each of those time points occurred during stages of the pandemic where rates of infection and related restrictions were *not* at their peak (specifics vary across UK nations and, indeed, regions, but broadly follow a similar trend in these time points).^22–24^ In July 2020, the first substantial wave of COVID-19 infections was generally considered to have ended,^23^ restrictions had been eased (to varying extents) across much of the UK, and many school-age adolescents were attending school, even if only to finish the academic year after long absences. In November 2020, a second wave had begun, and rates were increasing, but restrictions were still relaxed (comparative to previous restrictions), and most school-age adolescents were attending school (except in Wales, which implemented ‘firebreak’ school closures).^22^ In March 2021, many school-age adolescents were returning to schools across the UK following closures.^22,24–26^ The SDQ measure used asks respondents to reflect on the last six months, and so does cover peak periods; however, literature has highlighted recall bias as prevalent in measures reliant on such recall, particularly among children and adolescents whose episodic thinking is still developing.^27^ Given this, it is difficult to know for certain the extent to which our data can be considered a reflection of the overall pandemic period or of these non-peak periods. There is some evidence that adolescent mental health symptoms shift in line with pandemic changes, with heightened reporting during periods of more restrictive lockdown measures.^28^ Thus, although we cannot say with certainty, it seems likely that had the participants been surveyed in the peak of COVID-19-related restrictions, their responses may have differed. However, it is still valuable to understand how mental health patterns changed during these periods, where there was still a high level of uncertainty and worry for many. A final consideration is that there may be longer-term effects of the pandemic that are not captured by the current study – for example, there may be children who were very young during the pandemic who were affected by the lack of social contact.

Indications of continuity in pre-pandemic mental health trends raise considerations for how we conceptualise effects of the pandemic on youth mental health. It has been argued that indications of short-term impact on young people (and the wider population) reflect not a worsening of mental health but healthy, normative, emotional reactions to profound disruption to everyday life, and that we must take care not to pathologise this as a crisis for most young people.^29,30^ It may well be that such emotional reactions are short-lived, context-specific or do not, in the longer term, translate to items targeting general mental health symptomatology (of course, our examination of mental health here is not exhaustive and does not, for instance, cover eating disorders). Similarly, there are questions as to whether we have underestimated our capacity for adaptation in the face of crisis; insights from global resilience research have demonstrated the capacity for multilevel adaptation in the face of disaster across individuals, communities and complex systems.^31^ Indeed, several studies have highlighted various adaptive processes among adolescents in the context of lockdowns.^32,33^ In short, the pandemic may not have affected adolescent mental health as significantly in respect to widespread mental health harm as initially feared. Indeed, our longitudinal findings would suggest a general resilience among youth, an important finding highlighting how adolescents were able to cope with the implications of the pandemic.

### Improvement in conduct problems

Contrary to our hypothesis, significant change over time was identified for conduct problems, which *improved* slightly; that is, adolescents reported *fewer* conduct problems in the context of the pandemic than they had previously. The effect size observed here (β = −0.26) is modest but meaningful considering typical effect sizes in this area and the longitudinal nature of data, where effect sizes are often smaller.^34^ This finding contradicts evidence showing increased conduct problems during the pandemic.^16^ However, such evidence is from the very early stages of the pandemic, prior to or during July 2020, where mental health effects may have been different, and may be affected by frequently observed sampling problems. Though we cannot offer conclusive explanations, there are potential ones. To aid reader interpretation, the subscale items are: *I get very angry and often lose my temper; I usually do as I am told; I fight a lot. I can make other people do what I want; I am often accused of lying or cheating; I take things that are not mine from home, school or elsewhere.* One consideration may that in completing our surveys, some adolescents were reflecting on time spent at home rather than school, while others were reflecting on school experiences during an unusual period. For those thinking about recent time at home, it is possible that for many this time was less strictly managed, with different rules and routines to the structured time that adolescents typically experience in daily school life and, perhaps, less active supervision. Challenges around disobedience and fighting *may* therefore be less pronounced in the home, or perhaps only *perceived* to be less pronounced by an adolescent self-reporting. Conversely, for those at school, class sizes were often considerably smaller than usual (advised to be less than half the size wherever possible^35^), and educator guidance included an emphasis on flexibility and patience around behaviour.^36^ It may be that in this atmosphere adolescents were less inclined to such behaviours, or may have*perceived* themselves as engaging in less of this behaviour within more relaxed rules or smaller groups. Overall, this finding is particularly pertinent amid ongoing efforts to understand key trends such as growing rates of school suspension and exclusion following pandemic restrictions, and whether these link to behavioural issues or changes in practice (e.g.^37^). Ongoing research using multiple methods to examine this outcome across later points in the pandemic and in the context of school closures and re-openings would be valuable.

### Homogeneity of results across groups

A surprising finding is the lack of significant group differences across changes to total mental health difficulties associated with pandemic surveys. There was widespread concern that there would be disproportionate pandemic mental health effects, and a number of studies reported greater impact for some groups, such as girls or young women^12^ and children from more deprived households.^38^ However, we note that another study did not find any heterogeneity in the responses to the pandemic.^13^ Our findings may also reflect the specificity of impact for particular groups; for instance, evidence does suggest that facets of socioeconomic disadvantage were associated with worsened mental health among adults during the pandemic, but perhaps such challenges had fewer direct implications for mental health among the majority of adolescents.^39^ There were some groups that we could not examine, because of lack of available data, for whom this might not have been the case – for example, those with prior mental illness, special educational needs or neurodevelopmental disorders – as there is evidence of impact on these groups more widely (e.g.^16^). This may also differ from some of the characteristics we examined here, such as deprivation, in being more related to one’s health or conditions relative to the wider socioeconomic context.

### Strengths and limitations

The current study offers several strengths, including our use of self-reported mental health data, modelling of pre-pandemic trends and sample representativeness in the (weighted) data. However, we also acknowledge various limitations. As explored earlier, the time points of our data map on to less intense periods of the first year of the pandemic, avoiding peaks of infections and the height(s) of restrictions, and tend to be near the beginning and end of substantive school closures or the strictest social-distancing measures. This is a strength in that it allows us to ‘zoom out’ beyond particularly disruptive periods that may prompt more immediate emotional reactivity; however, our lack of data in this sample for such periods limits our ability to understand more subtle shifts across peaks and troughs of the pandemic. Our study offers important, rigorous findings of trends across this period, which should be interpreted carefully in the context of ongoing shifts in discourses linked to the pandemic.

We also highlight measurement issues; though the SDQ has demonstrated acceptable reliability and criterion validity, this measure has been critiqued for issues including readability and age-appropriateness,^40^ and it is also particularly brief with limited response options. Future research would benefit from more comprehensive mental health assessments and continued triangulation across varying measurement approaches. We note that the questionnaire did not include the ‘impact supplement’, which assesses functional impairment from symptoms across an individual’s life. Though this was not problematic, as we were interested in impact on mental health difficulties rather than the functional impairment incurred through such difficulties, building an understanding of this using other data-sets can support understanding of the implications of mental health difficulties, specifically in the COVID-19 context. Finally, the study sample has a substantial proportion of non-response, which was higher during the pandemic than before it. This raises the possibility of bias if the factors influencing non-response are related to the outcome, mental health. To mitigate this, survey weights were calculated based on area-level characteristics of the sample, and the weighted sample characteristics were found to be similar before and after the pandemic. These weights should adequately address the potential bias unless there is an unaccounted-for relationship between non-response and mental health. For instance, if households with single parents were less likely to respond during the pandemic and were also more affected by it, the weighted results may still be biased. This is because single-parent status was not included in the weighting model, meaning any systematic differences related to this characteristic would remain uncorrected.

Overall, the current study offers evidence, drawn from a representative sample, that youth mental health did not deteriorate over time across three months of the pandemic. This finding is at least applicable to those aspects of mental health measured by the SDQ. This is an important contribution to the complex evidence base as we continue to unpack the mental health implications of the pandemic, and it may support the idea that most young people’s emotional responses to the pandemic were represented by normative and short-lived changes, rather than a widespread and ongoing public health crisis. Evidence of a reduction, albeit a modest one, in self-reported conduct problems is of interest, and should prompt further exploration of the ways that behaviour was affected or responded to in the context of the first year of the pandemic, and potential shifts and implications over time (e.g. implications upon returning to school). Our research also highlights the importance of monitoring overall trends, including in representative sampling, and of not ignoring the focus on pre- and, indeed, post-pandemic worsening trends: these must continue to be given dedicated attention. Even prior to the pandemic, the mental health of adolescents was deteriorating;^17^ budget cuts and wider challenges meant that mental health provision, including that offered in schools, was described as being at ‘breaking point’.^41^ These examples of an already existing escalation of mental health difficulties situate the current study as a further contribution to an evidence base documenting shifts (or lack thereof) at particular points in time. ‘Crisis’ has served as an ongoing narrative in young people’s mental health for several years, and this was further amplified in relation to the pandemic. As we move further away from major restrictions linked to the pandemic in the UK, a continued measured response by researchers is necessary to establishing the extent of the pandemic’s impact on young people’s mental health and, in turn, appropriate policy and practice contextualisation and responses to rates of difficulties.

## Supporting information

Pierce et al. supplementary material Pierce et al. supplementary material

## Data Availability

The data that support the findings of this study are openly available via the UK Data Service Repository.

## Footnotes

a. Our gender variable here relies on an imperfect proxy by drawing on self-reported binary sex data; however, in the absence of more inclusive gender data we sought to be sensitive to the ethical ramifications of implying attributions to sex and biological difference.

## Supplementary material

The supplementary material is available online at https://doi.org/10.1192/bjo.2025.10801
