# Supplementary material for: Changes in mental health of adolescents during three months of the COVID-19 pandemic: longitudinal study
Source: BJPsych Open. 2025 Aug 15;11(5):e177. doi: 10.1192/bjo.2025.10801 (PMC12451728; doi:10.1192/bjo.2025.10801)
Supplement: Pierce et al. supplementary material [file S2056472425108016sup001.docx]

**Supplementary Materials**

Supplementary materials include the following appendices: A) Description of missing data; B) Data analysis plan; C) Psychometric properties of the SDQ during the pandemic; and D) Overview of timeframes, completeness of measures, and unweighted and weighted SDQ scores;

**Appendix A: description of missing data**

Number and proportion of responses with missing data for analysis variables

| Variable | N | % |
| --- | --- | --- |
| Age | 90 | 0.7 |
| Gender | 31 | 0.2 |
| SDQ emotional subscale | 62 | 0.5 |
| SDQ conduct subscale | 56 | 0.4 |
| SDQ hyperactivity subscale | 65 | 0.5 |
| SDQ peer problems subscale | 54 | 0.4 |
| SDQ prosocial subscale | 51 | 0.4 |
| Urban/rural indicator | 116 | 0.9 |
| Income | 421 | 3.3 |
| Age | 90 | 0.7 |
| Ethnicity | 1097 | 8.6 |
| Region | 77 | 0.6 |
| Any | 1,573 | 12.3 |

**Appendix B) Data analysis plan**

**Analysis plan finalised in agreement between Authors MPa, EB, SG, and OD prior to analysis**

**Background:** The early phases of the pandemic led to an increase in mental distress among UK adults. The effect on children and young people’s mental health is less clear.

**Primary aim:** to examine how young people’s self-reported mental health changed during the pandemic.

**Secondary aims:** To examine if the pandemic had a differential effect according to: different dimensions of young people’s mental health; different phases of the pandemic; and for different subgroups by age, gender, ethnicity and deprivation.

**Hypotheses:** 1. the pandemic led to a deterioration in young people’s expected mental health; 2. The effect was greatest during the early phases of the pandemic; for girls; those in deprived households; those from ethnic minority groups; and those in northern regions.

**Sample:** young people, aged 10-15, who answered at least one COVID-19 survey as part of the ‘Understanding Society’ survey, in one of three waves: July 2020, November 2020, or March 2021. Data will also be extracted on young people surveyed in the main UKHLS waves 9 and 11, conducted Jan 2017 to Mar 2019 and Jan 2019 to May 2021 respectively.

**Primary outcome variables:** The total score from the strengths and difficulties questionnaire (SDQ).

**Secondary outcome variables:** subscales of the SDQ: prosocial; hyperactivity; emotional; peer problems; conduct problems.

**Covariates:** gender, age at interview, ethnicity (Asian, Black, Mixed, White, Other), region of the UK (Northern England, Midlands, Southern England, Wales, Scotland, Northern Ireland), quintile of household income (measured pre-pandemic).

**Analysis:**

Graphical representations of repeated cross-sectional data will be done for the whole sample, then by SDQ domains, separately by boys and girls. Then random effects models will be fitted to the sample, to examine whether the COVID-19 pandemic was associated with a greater than expected increase in symptoms of mental distress. These models will control for calendar time (parameterised as years since first interview in the sample) and calendar time squared, and an interaction between calendar time and gender. They will include longitudinal weights, provided by UKHLS.

Models will be refitted to examine the effect on domains of the SDQ, separately by gender. Then interactions will be fitted to examine change in overall SDQ associated with the pandemic within subgroups: gender, age (10-13, 14-15), ethnicity, region of the UK, quintile of household deprivation, period of the pandemic (July, November, or March 2021). P-values of less than .01 will indicate different effects according to subgroups.

**Appendix C: Psychometric properties of the SDQ during the pandemic**

*Methods*

Longitudinal measurement invariance was tested using 971 adolescents who responded to both the last pre-pandemic wave (Wave 11) and the first COVID wave (July 2020). Following the methods in Liu et el for testing longitudinal measurement invariance in ordered categorical data[1] , the longitudinal invariance structure was tested using different invariance models, from least to most restrictive:

- 1. Baseline: freely estimated factor loadings, thresholds and variances
  2. Factor invariance (A plus same factor loadings)
  3. Threshold invariance (B plus same threshold values)
  4. Unique factor invariance – (C plus unique factor variances are equal over time)

For each model, fit was determined using the Comparative Fit Index (CFI), where values over 0.95 are considered good fit, and the RMSEA, where good fit is considered for values under 0.05. The invariance structure was tested using the difference test, which compares the current model to the prior one. A p-value of less than 0.05 means there is sufficient evidence to suggest the current model does not fit the data any better than the previous one.

***Longitudinal factor analysis 1: Emotional scale***

|  | **CFI** | **RMSEA** | **Difference test** | | |
| --- | --- | --- | --- | --- | --- |
| **Model** |  |  | **χ^2^** | **dof** | **p-value** |
| Baseline | 0.978 | 0.060 | - | | |
| Factor invariance | 0.979 | 0.054 | 3.009 | 4 | 0.5564 |
| Threshold invariance | 0.982 | 0.048 | 3.865 | 4 | 0.4245 |
| Unique factor invariance | 0.985 | 0.042 | 5.259 | 5 | 0.3851 |

***Longitudinal factor analysis 2: Hyperactivity scale***

|  | **CFI** | **RMSEA** | **Diff-test** | | |
| --- | --- | --- | --- | --- | --- |
| **Model** |  |  | **χ^2^** | **dof** | **p-value** |
| Baseline | 0.952 | 0.088 | - | | |
| Factor invariance | 0.948 | 0.086 | 29.269 | 4 | <0.0001 |

***Longitudinal factor analysis 3: Conduct scale***

|  | **CFI** | **RMSEA** | **Diff-test** | | |
| --- | --- | --- | --- | --- | --- |
| **Model** |  |  | **χ^2^** | **dof** | **p-value** |
| Baseline | 0.996 | 0.015 |  | | |
| Factor invariance | 0.998 | 0.009 | 1.975 | 4 | 0.7404 |
| Threshold invariance | 0.998 | 0.009 | 4.338 | 4 | 0.3622 |
| Unique factor invariance | 0.995 | 0.014 | 8.382 | 5 | 0.1364 |

***Longitudinal factor analysis 4: Peer problems scale***

|  | **CFI** | **RMSEA** | **Diff-test** | | |
| --- | --- | --- | --- | --- | --- |
| **Model** |  |  | **χ^2^** | **dof** | **p-value** |
| Baseline | 0.956 | 0.049 |  | | |
| Factor invariance | 0.958 | 0.045 | 3.948 | 4 | 0.4131 |
| Threshold invariance | 0.959 | 0.042 | 6.375 | 4 | 0.1729 |
| Unique factor invariance | 0.940 | 0.048 | 30.557 | 5 | <0.0001 |

***Longitudinal factor analysis 5: Prosocial scale***

|  | **CFI** | **RMSEA** | **Diff-test** | | |
| --- | --- | --- | --- | --- | --- |
| **Model** |  |  | **χ^2^** | **dof** | **p-value** |
| Baseline | 0.995 | 0.019 |  | | |
| Factor invariance | 0.997 | 0.013 | 2.968 | 4 | 0.5632 |
| Threshold invariance | 0.996 | 0.016 | 8.125 | 4 | 0.0871 |
| Unique factor invariance | 0.998 | 0.010 | 4.314 | 5 | 0.5051 |

1 Liu Y, Millsap RE, West SG, *et al.* Testing measurement invariance in longitudinal data with ordered-categorical measures. *Psychol Methods* 2017;**22**:486–506. doi:10.1037/met0000075

**Appendix D: Overview of timeframes, completeness of measures, and unweighted and weighted SDQ scores**

| **Timeframes, completeness of measures, and unweighted and weighted SDQ scores** | | | | | | |  |
| --- | --- | --- | --- | --- | --- | --- | --- |
| **Statistic** |  | **Main survey** | | **COVID surveys** | | | |
|  | Wave 7 | Wave 9 | Wave 11 | 1 | 2 | 3 | |
| Dates of data collection | Jan 2015 to Apr 2017 | Jan 2017 to May 2019 | Jan 2019 to May 2021 | Jul 2020 | Nov 2020 | Mar 2021 | |
| Sample size | 3,629 | 2,821 | 2,100 | 1,411 | 1,432 | 1,388 | |
| Complete SDQ data | 3,606 | 2,791 | 2,093 | 1,407 | 1,423 | 1,386 | |
| **Mean SDQ scores (SD), unweighted** | | | | | | |  |
| Total | 10.7 (5.8) | 10.7 (5.9) | 11.3 (6.1) | 10.9 (6.0) | 11.4 (6.3) | 11.1 (6.1) | |
| Emotional | 2.9 (2.3) | 3.0 (2.4) | 3.2 (2.5) | 3.1 (2.4) | 3.4 (2.5) | 3.2 (2.5) | |
| Conduct | 2.0 (1.7) | 1.9 (1.7) | 2.0 (1.7) | 1.8 (1.7) | 1.8 (1.7) | 1.7 (1.6) | |
| Hyperactivity | 3.8 (2.3) | 3.9 (2.4) | 4.0 (2.4) | 4.0 (2.4) | 4.2 (2.6) | 4.2 (2.5) | |
| Peer | 1.9 (1.7) | 1.9 (1.8) | 2.0 (1.7) | 2.1 (1.7) | 2.0 (1.7) | 2.0 (1.7) | |
| Prosocial | 7.8 (1.8) | 7.8 (1.8) | 7.7 (1.8) | 7.9 (1.7) | 7.7 (1.7) | 7.8 (1.8) | |
| **Mean SDQ scores (SD), weighted** | | | | | | |  |
| Total | 11.1 (6.0) | 11.4 (6.0) | 11.8 (6.1) | 11.9 (6.1) | 11.9 (6.3) | 11.4 (6.3) | |
| Emotional | 3.1 (2.4) | 3.2 (2.4) | 3.4 (2.5) | 3.4 (2.5) | 3.6 (2.6) | 3.2 (2.5) | |
| Conduct | 2.1 (1.8) | 2.1 (1.7) | 2.1 (1.8) | 1.9 (1.7) | 1.9 (1.7) | 1.8 (1.7) | |
| Hyperactivity | 4.0 (2.4) | 4.1 (2.4) | 4.2 (2.4) | 4.2 (2.4) | 4.3 (2.6) | 4.3 (2.6) | |
| Peer | 1.9 (1.8) | 2.0 (1.8) | 2.1 (1.8) | 2.3 (1.9) | 2.1 (1.8) | 2.1 (1.8) | |
| Prosocial | 7.8 (1.8) | 7.8 (1.7) | 7.7 (1.8) | 7.8 (1.8) | 7.6 (1.7) | 7.8 (1.8) | |
| SDQ = Strengths and Difficulties Questionnaire | | | | | | |  |
